# Sizing down and functionalizing polylactide (PLA) resin for synthesis of PLA-based polyurethanes for use in biomedical applications

**DOI:** 10.1038/s41598-023-29496-x

**Published:** 2023-02-09

**Authors:** Bunthoeun Nim, Sosna Sri Rahayu, Kamonchanok Thananukul, Chorney Eang, Mantana Opaprakasit, Atitsa Petchsuk, Chariya Kaewsaneha, Duangporn Polpanich, Pakorn Opaprakasit

**Affiliations:** 1 School of Integrated Science and Innovation, Sirindhorn International Institute of Technology (SIIT), Thammasat University, Pathum Thani, 12121 Thailand; 2 Department of Materials Science, Faculty of Science, Chulalongkorn University, Bangkok, 10330 Thailand; 3 National Metal and Materials Technology Center (MTEC), National Science and Technology Development Agency (NSTDA), Thailand Science Park, Pathum Thani, 12120 Thailand; 4 National Nanotechnology Center (NANOTEC), National Science and Technology Development Agency (NSTDA), Thailand Science Park, Pathum Thani, 12120 Thailand

**Keywords:** Chemistry, Materials science

## Abstract

Alcoholysis is a promising approach for upcycling postconsumer polylactide (PLA) products into valuable constituents. In addition, an alcohol-acidolysis of PLA by multifunctional 2,2-bis(hydroxymethyl)propionic acid (DMPA) produces lactate oligomers with hydroxyl and carboxylic acid terminals. In this work, a process for sizing down commercial PLA resin to optimum medium-sized lactate oligomers is developed at a lower cost than a bottom-up synthesis from its monomer. The microwave-assisted reaction is conveniently conducted at 220–240 °C and pressure lower than 100 psi. The PLA resin was completely converted via alcohol-acidolysis reaction, with a product purification yield as high as 93%. The resulting products are characterized by FTIR, 2D-NMR, ^1^H-NMR, GPC, DSC, and XRD spectroscopy. The effects of PLA: DMPA feed ratios and the incorporation of 1,4-butanediol (BDO) on the structures, properties, and particle formability of the alcohol-acidolyzed products are examined. The products from a ratio of 12:1, which possessed optimum size and structures, are used to synthesize PLA-based polyurethane (PUD) by reacting with 1,6-diisocyanatohexane (HDI). The resulting PUD is employed in encapsulating lavender essential oil (LO). Without using any surfactant, stable LO-loaded nanoparticles are prepared due to the copolymer’s self-stabilizability from its carboxylate groups. The effect of the polymer: LO feed ratio (1.25–3.75: 1) on the physicochemical properties of the resulting nanoparticles, e.g., colloidal stability (zeta potential > -60 mV), hydrodynamic size (300–500 nm), encapsulation efficiency (80–88%), and in vitro release, are investigated. The LO-loaded nanoparticles show non-toxicity to fibroblast cells, with an IC_50_ value higher than 2000 µg/mL. The products from this process have high potential as drug encapsulation templates in biomedical applications.

## Introduction

Polylactide (PLA) and its derivatives, especially those fabricated as nano- or microparticles and fibers, have attracted vast attention in medical, pharmaceutical, and cosmetic applications^1–3^. The materials have excellent biocompatibility, absorbability, and degradability with high cell affinity/immunogenicity. In addition, these can be hydrolyzed and metabolized into non-toxic compounds after applications^4^, especially as controlled-release materials. PLA-based polymeric particles have been widely used in the encapsulation and control release of various drugs, active compounds, and bioactive cells. These particles were specifically used to prevent drug degradation during the delivery process to the target cells^1,5,6^. The materials were employed in oral drug delivery to improve the efficiency of reaching systemic circulation with reduced gastric toxicity^7^. Various bioactive molecules or drugs have been successfully encapsulated into micro- or nanospheres of PLA derivatives, such as melittin^8^, metformin hydrochloride^9^, ibuprofen^7,10^, paclitaxel^11,12^, amoxicillin^13^, anti-HIV drugs^14^, insulin^15,16^, plasmid DNA^17,18^, and various proteins^19–21^.

Alcoholysis has been employed in the chemical recycling of postconsumer PLA products to medium or small-sized lactate sequences using various alcohols and polyols, which are then used as starting materials in synthesizing other value-added (co)polymers^22–30^. The process can be applied to virgin PLA resin for sizing down and functionalizing commercial PLA to medium-sized products with unique functional groups suitable for specific applications, e.g., food-contact packaging and cosmetics. These provide high-purity products, with easy processing and lower-price starting materials, compared to those synthesized from bottom-up processes using lactic acid or lactide. The major products of the alcoholysis process are hydroxyl-terminated lactates, which can be used as solvents or starting materials for other degradable polymers^29–32^. Furthermore, the multi-hydroxyl products are promising as hydroxyl-containing materials in various uses, such as compatibilizers, additives, adhesives, and plasticizers. Elastomeric PLA-based polyurethanes (PUs) have been prepared from the reactions between alcoholized PLA-diols with 1,6-diisocyanatohexane (HDI). The PU materials have high elasticity with low modulus and adequate strength compared to neat PLA^29,30^, which are suitable for use as single-component functional materials or additives to improve the mechanical properties of other bioplastics through blending processes^33^. In contrast, PLA-based PUs synthesized from 4,4-diphenylmethane diisocyanate (MDI) or toluene 2,4-diisocyanate (TDI) exhibited improved thermal and mechanical properties with moderate flexibility^34^.

Degradable multifunctional and hyperbranched polymeric materials have caught vast attention in various fields ranging from biomedical materials, adhesives, hydrogels, and waterborne applications. This pertains to their excellent physical and chemical properties in hydrophilicity, cell compatibility, biodegradability, self-emulsification, self-stabilization, non-toxicity, and non-inflammatory^35–37^. These can be achieved by incorporating multifunctional monomers, chain extenders, initiators, and cross-linkers. For instance, non-toxic PUs derived from bio-based cellulose and ʟ-Lysine diisocyanate ethyl ester (ʟ-LDI) exhibited biodegradability in both hydrolytic and enzymatic media^38^. Selected PLA-based PUs possessed high biodegradability in the presence of phosphate-buffer saline solution and enzyme medium^34^. 2,2-bis(hydroxymethyl)propionic acid (DMPA) was used as a monomer, chain extender, or internal emulsifier by incorporating its multi-hydrophilic groups (hydroxyl and carboxylic acid) into the polymer backbone, leading to enhanced self-stabilizability in dispersion or emulsion systems^39,40^. DMPA has been used in preparing waterborne PU dispersions^41,42^, PU ionomers^40^, water-soluble copolymers^43^, water-dispersable polymers^44^, coatings^45^, adhesives^46^, and water-based ink binders^47^. DMPA plays a role as an anionic constituent due to the presence of a carboxylic acid group, which can be converted to carboxylate^48^. The compound acts as an ionic self-emulsifying unit for stabilizing the polymer dispersion particles in an aqueous medium and reducing the particle size due to enhanced hydrophilicity^49^. The incorporation of neat PLA with quaternized soybean oil generated degradable materials with antimicrobial properties. These exhibited a higher degradation rate in the presence of lipase enzyme than that of hydrolytic medium^4^. The enzymatic degradation rates of neat PLA and selected PLA-based PUs were compared. The results showed a similar rate, indicating that the degradation mechanisms mainly proceed through surface erosion than inner chain degradation^34,50^.

Given the versatility of polyfunctional and hyperbranched materials, converting PLA to multifunctional copolymers by a chemical recycling process is a promising approach for turning rigid and brittle PLA waste into valuable products. The alcoholysis of PLA by various hydroxyl-containing reagents has been reported to recover functionalized lactates, which can be used as building blocks for other useful degradable copolymers^51^. These can be in linear, branched, or network structures, depending on the number of hydroxyl terminals of the alcohol reagents employed for cleaving the ester bonds of PLA^52^. PLA-diols and polyols were obtained from the alcoholysis of PLA with di-hydroxyl and poly-hydroxyl alcohols^26,27,53^. Introducing anionic functionals onto the branched chains of lactate structures can broaden the material applications due to increasing hydrophilicity surface^54–58^. High-MW PLA can be cleaved by multifunctional reagents comprising hydroxyls and carboxylic acid groups, *e,g.*, DMPA, citric acid, tartaric acid, and glutaric acid. The ester bonds of PLA are cleaved by hydroxyl groups via alcoholysis, while the free carboxylic acid functions as anionic centers. In addition, when the reaction is carried out at extreme conditions of high temperature (> T_m_ of PLA) and high pressure, the ester bonds can be cleaved via acidolysis reaction through exchangeable reaction with carboxylic acids of the reagents, generating carboxylic terminal groups.

Various preparation techniques have been developed for producing nanoparticles from degradable/biocompatible polymers, especially PLA and derivatives, for drug encapsulation and controlled-release applications. These include emulsion (single and double emulsion)^59–64^, precipitation^1^, solvent extraction/evaporation^11^, suspension ^65,66^, and spray drying^12^. The choice of these techniques depends on the physicochemical properties of the encapsulated drugs, the selected polymer template (degradation rate, morphology, particle-sized distribution), the site-specific delivery of the drug, and the duration of the therapeutic action^67–70^. Nanoemulsion with submicron-sized droplets (approximately 10 to 1000 nm in diameter) is a versatile system for preparing nanoparticles. An oil-in-water (O/W) nanoemulsion technique is effective and convenient for encapsulating and protecting hydrophobic drugs from environmental degradations, e.g., hydrolysis and oxidation^71–73^*.* Furthermore, nano-emulsification improves the drug's efficacy, reducing the required dose, minimizing the side effects, and increasing its solubility and bioavailability^74,75^. Drug-encapsulated polymeric nanoparticles are commonly prepared via an O/W nanoemulsion by mixing the dispersed oil phase, which contains hydrophobic drugs and polymer molecules, and the continuous aqueous phase, with the application of a high shear force. Stable oil droplets are well dispersed in the aqueous phase with the support of stabilizing agents. The molecules are located at the droplet interface, stabilizing the system and preventing phase separation. After solvent removal, the stable solid nanoparticles of hydrophobic drugs entrapped in a polymer matrix are produced.

The use of alternative medicines, especially medicinal herbs, for treating various conditions is increasing. The interest in their potential applications has grown all over the world. *Lavandula angustifolia* (Lavender) essential oil (LO), consisting of different compounds like linalool and linalyl acetate, is of interest as a medicinal herb due to various excellent properties, e.g., analgesic, anti-inflammatory, antioxidant, antibacterial, antifungal, sedative, and antidepressant effects, which can effectively heal burns^75,76^. It is widely used in biomedical applications to help the skin's endogenous protection system from oxidative damage. However, LO is volatile and unstable under ambient conditions caused by light, air, temperature, and moisture^75,77,78^. The compounds could be easily degraded if not properly protected. Therefore, encapsulation technology is necessary to improve the stability of LO, provide a controlled release, and increase an action duration. Kazemi et al*.* prepared a nanoemulsion of LO and licorice extract for skin wound healing^75^. The oils were mixed with tween20 and tween80 surfactants. After emulsification with an aqueous phase containing a co-solvent of glycerin and polyethylene glycol 400 under heat stirring, nanoemulsion creams were produced. The nanoemulsion creams were then used to treat deep skin wounds of rat models. The results demonstrated that LO-based nanoemulsion cream improved the wound healing efficiency in different stages, *i.e.*, wound closure, epithelialization, and molecular processes, including the increase in expression of TGF-β1, type I, and type III collagen genes.

**Figure 1:**
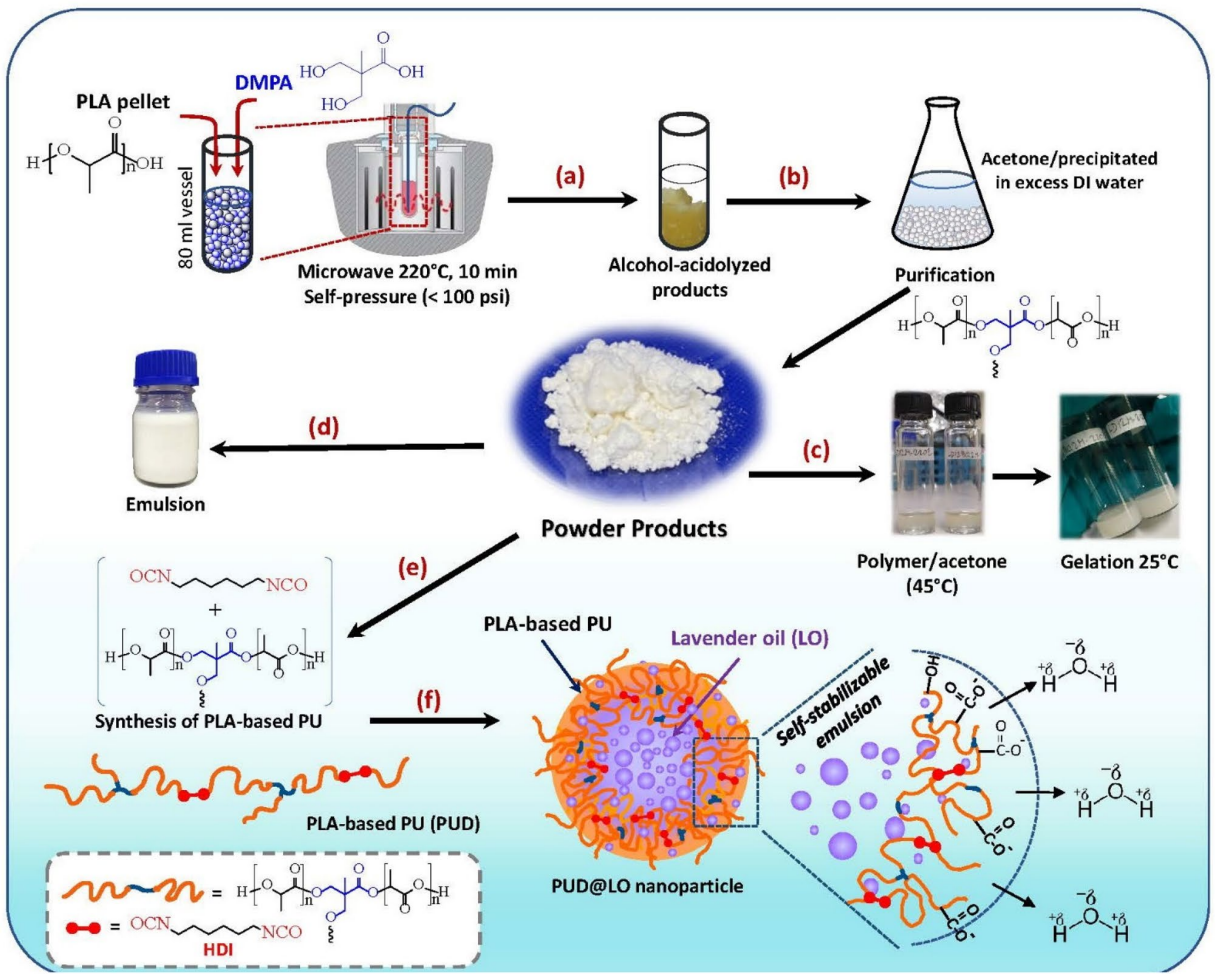
Overview of a process for sizing down and functionalizing PLA resin for preparing carboxylate-containing PLA-based polyurethanes for use as self-stabilizable emulsion and encapsulation of lavender oil.

In this work, a process for sizing down commercial PLA resin to optimum medium-sized lactate oligomers containing hydroxyl and carboxylic acid terminals is developed, as summarized in Fig. 1. An alcohol-acidolysis or transesterification of PLA by 2,2-bis(hydroxymethyl) propionic acid (DMPA) is employed using microwave irradiation. The process is conveniently conducted at a lower cost than a bottom-up synthesis from lactic acid monomer or lactide. The effects of the PLA: DMPA feed ratio and the incorporation of 1,4-butanediol (BDO) on the structures, properties, and particle formability of the alcohol-acidolyzed products are examined. The resulting products, with optimum molecular weight and chemical structure, are used to synthesize PLA-based polyurethane (PUD) by reacting with HDI, whose structures and properties are assessed. The linear HDI is purposely employed to synthesize elastomeric PU comprising excellent biocompatibility and biodegradable properties, with non-toxic degradation by-products, which are essential for biomedical applications^79–82^. The resulting PUD is employed in encapsulating lavender essential oil (LO) via an O/W nanoemulsion/solvent evaporation technique. Without using any surfactant, stable LO-loaded nanoparticles can be prepared. The effect of the polymer: LO feed ratio on the physicochemical properties of the resulting nanoparticles, e.g., colloidal stability, hydrodynamic size, polydispersity index, encapsulation efficiency, and in vitro release, are investigated. The toxicity of the LO-loaded nanoparticles to fibroblast cells is assessed. The products from this process have high potential in biomedical applications.

## Experimental

### Materials

Polylactide 4043D (PLA) was purchased from NatureWorks. 1,4-butanediol (BDO) (> 99%), 1,6-diisocyanatohexane (HDI) (> 99%), and 2,2-bis(hydroxymethyl)propionic acid (DMPA) (98%) were obtained from Acros Organics. Tin octoate, Sn(Oct)_2_, was purchased from Wako. Sodium dodecyl sulfate (SDS) was provided by Carlo Erba Reagent. Sodium hydroxide anhydrous, hydrochloric acid, dichloromethane (DCM), chloroform, and acetone were purchased from Carlo Erba. Lavender oil was obtained from Thai-China Flavours and Fragrances Industry Ltd. All chemicals were used without further purification. Deionized (DI) water was used throughout this work.

### Characterizations

The chemical structures of the alcohol-acidolyzed PLA products were characterized by Fourier transform infrared (FTIR) spectroscopy in an attenuated total reflectance (ATR) mode on a Nicolet iS5 spectrometer (Thermo Scientific, USA), proton nuclear magnetic resonance (^1^H-NMR) spectroscopy (600 MHz, Bruker, Germany), X-ray diffraction (XRD) spectroscopy on an X-ray diffractometer (D8 Advance, Bruker, Germany) using CuKα radiation (1.5406 Å) at 40 kV and 40 mA, and two-dimensional NMR (2D-NMR) spectroscopy, based on heteronuclear multiple quantum correlation (HMQC) and heteronuclear multiple-bond coherence (HMBC) techniques on an Ascend TM 600 Bruker spectrometer. The thermal properties of the products were examined by differential scanning calorimetry (DSC) on a DSC822e (Mettler Toledo, Switzerland) under a nitrogen atmosphere (a flow rate of 60 mL/min) and a heating/cooling rate of 10 °C/min. The material’s morphology was observed on a field-emission scanning electron microscope (FE-SEM) (JEOL JSM7800F, Japan) with an acceleration voltage of 1 kV at different magnifications. The particle stability in an aqueous medium was examined in terms of Zeta potential on a Malvern Zetasizer Nano-ZS (Malvern, United Kingdom).

### Alcohol-acidolysis of PLA

Alcohol-acidolyzed PLA products were synthesized from commercial PLA resin (${\overline{M} }_{w}$= 1.2–1.5 × 10^5^ g/mol) to medium-sized oligomers with hydroxyl and carboxylic acid terminals, as illustrated in Fig. 1a. The reaction was conducted in a microwave reactor (Discover SP series, CEM Mattews NC, USA) at 220–240 °C under self-developing pressure (< 100 psi) for 10 min. The PLA: DMPA feed ratio was varied from 6:1, 12:1 to 16:1 wt/wt. The corresponding reactions by a mixture of DMPA/BDO were also conducted at PLA: DMPA: BDO feed ratios of 12: 0.8: 0.2 and 16: 0.8: 0.2. The samples are coded according to the feed ratio, e.g., LD12BD0.2 M is derived from the 12: 0.8: 0.2 ratio. As solid residue of unreacted PLA was not observed after the reaction, this indicates a complete conversion of PLA to its corresponding oligomers. The products were dried at 60 °C in an oven overnight. The overall experimental conditions are summarized in Supplemental Table S1. All products were then purified via a precipitation technique, as shown in Fig. 1b. The samples were dissolved in a minimum amount of acetone at 40–50 °C, followed by precipitating in an excess amount of DI water. Finally, the alcohol-acidolyzed PLA particles were vacuum filtered and dried in an oven at 60 °C overnight. The yields of the purified products ranged from 82.6 to 93.2% (Supplemental Table S1). The white powder products can be redissolved in acetone and turned into gel at room temperature, as shown in Fig. 1c. The supernatant containing water and acetone-soluble molecules was vacuum dried at 100 °C. The resulting solid products were further characterized for appropriate applications.

### PLA-based polyurethane from alcohol-acidolyzed PLA

PLA-based polyurethane (PU) is synthesized from the resulting alcohol-acidolyzed PLA products by reacting with HDI, as shown in Fig. 1e. LD12M was chosen as an optimum representative due to its small average particle size, narrow polydispersity index (PDI), and large Zeta potential value. LD12M (3 g) with 0.5 wt% of tin octoate, Sn(Oct)_2_, catalyst was dissolved in 40 ml of chloroform at 50 °C under a reflux condition. A varied amount of HDI was added to the oligomer solution under constant temperature for 5 h, according to the synthesis conditions as summarized in Supplemental Table S2, in which the sample codes are also described. The resulting PLA-based PU in CHCl_3_ solvent was directly used in preparing the oil/water emulsion and encapsulation of LO.

### Self-stabilizability of the products in emulsion systems

As the resulting alcohol-acidolyzed PLA contains hydroxyl and carboxylic acid terminals, which possess high negative polarity, their self-stabilizability in the emulsion process is assessed. The material was prepared as polymeric particles in an aqueous solution via emulsion/solvent evaporation. In Fig. 1d, the alcohol-acidolyzed PLA (6–12% w/v) was dissolved in dichloromethane (DCM, 5 mL) and transferred into an aqueous phase containing sodium dodecyl sulfate (SDS, 0.1% w/v). The oil-aqueous mixture was emulsified using a sonicator for 60 s at 50% amplitude. The emulsion was then continuously stirred at 500 rpm for 4 h to evaporate DCM. The effects of alkaline (NaOH) and acidic (HCl) media on particle stability were further examined. It is noted that the stable emulsion system can be directly prepared in alkaline pH without using SDS surfactant to improve particle stability in the aqueous medium. This is because the carboxylic acids are deprotonated by the NaOH solution. In contrast, the emulsion is unstable when the acid solution is used without adding an SDS surfactant. The effect of alkaline and acid contents on the morphology of the particles was examined by varying the pH ranges.

An oil/water emulsion of the synthesized PU/CHCl_3_ solution with an aqueous phase was also prepared to evaluate their self-stabilizability. PUD-0.5 solution (3 mL) was mixed with 50 mL SDS (0.1%) and sonicated for 60 s at 50% amplitude. In comparison, PU/CHCl_3_ solution (3 mL) was emulsified in alkaline (NaOH, 0.001 M) 50 mL for 60 s. The emulsions were stirred at 500 rpm overnight to evaporate the chloroform solvent.

### Encapsulation of lavender oil in PLA-based PU nanoparticles

Lavender essential oil (LO) was encapsulated into PLA-based PU (PUD) nanoparticles by O/W nanoemulsion/solvent evaporation technique. PUD-0.5 and LO were dissolved in chloroform at various PUD: LO wt. ratios, *i.e.*, 1.25:1, 2.5:1, and 3.75:1, as coded as PUD1.25@LO, PUD2.5@LO, and PUD3.75@LO. The oil phase was gently dispersed into an aqueous phase of 0.001 M NaOH (50 mL). PUD@LO nanoparticles were formed by Ultra-Turrax homogenizer (T25, IKA, Germany) at 10,000 rpm for 10 min. The solvent was evaporated in a fume hood for 24 h. The nanoparticles were purified by centrifugation and washed with DI water, as illustrated in Fig. 1f. The physicochemical characteristics of the nanoparticles, *i.e.*, hydrodynamic size, polydispersity index (PDI), zeta potential, encapsulation efficiency (%EE), loading capacity (%LC), and in vitro LO release behavior, were examined.

To determine the encapsulation efficiency (%EE) and loading content (%LC) of the encapsulated nanoparticles, the as-prepared suspension was centrifuged to separate the PUD@LO nanoparticles and free LO in the supernatant. The collected supernatant was analyzed using a UV–Vis spectrometer (Genesys 180, Thermo Fisher Scientific, USA) at 203 nm. The %EE and %LC values were calculated using Eqs. (1) and (2), respectively.

1 $$\% {\text{EE}} = \frac{{{\text{Initial amount of LO}} - {\text{Amount of LO in supernatant}}}}{{\text{Initial amount of LO}}} \times 100$$

2 $$\% {\text{LC}} = \frac{{{\text{Initial amount of LO}} - {\text{Amount of LO in supernatant}}}}{{\text{Weight of nanoparticles}}} \times 100$$

As PUD@LO cannot form a stable cast film, solid particles were separated by ultracentrifugation. After drying, the dried materials may be practically employed by embedding them in a film-forming polymer substrate, e.g., PVA, for wound healing or cell scaffold applications. The particles were dispersed in PVA films to determine the in vitro release of LO. PVA (MW 30,000–70,000 g/mol) solution in deionized water (2 wt.%, 2.5 mL) was prepared and cast using a silicone mold and dried for 1 h at 50 °C. The particles (25 mg) of each sample were embedded on the film’s surface at a ratio of 1:2 (w/w) and then air-dried overnight at room temperature to completely remove the solvent. The film samples (thickness of *ca.* 0.5–0.6 μm) were submerged in a phosphate buffer solution (PBS, pH 8.5), which is the pH of wound exudate at 37 °C ^83^. At different time intervals, the suspension was centrifuged, and 3 mL of the supernatant containing the released LO was taken and replaced by the same volume of fresh PBS. The collected samples were analyzed using UV–Vis spectroscopy (Genesys 180, Thermo Fisher Scientific, USA) at 203 nm.

### Cytotoxicity test

The cytotoxicity of PUD@LO nanoparticles was examined using an MTT cell proliferation assay. The HaCaT cell line (2 × 10^4^ cells/100 µL/well) was seeded in a 96-well microplate (Corning, USA) with a complete (10% FBS-supplemented) high-glucose DMEM medium (Gibco, USA). After that, the microplate was incubated in a 5% CO_2_/air incubator at 37 °C overnight to allow cell adhesion. The samples were dispersed in the complete DMEM at various concentrations (1–2000 μg/mL). Subsequently, 100 µL of each sample, including control (complete DMEM), were added to the cells seeded in a 96-well plate. After incubation for 24 h, the cells were washed with plain DMEM 2 times. The cell cytotoxicity was quantified using MTT assay, a colorimetric method, by adding 100 μl of MTT solution (5 mg/mL), then incubating at 37 ºC for 5 h in the dark. The plate was measured at 570 nm using a microplate reader.

## Results and discussion

### Chemical structures of alcohol-acidolyzed PLA products by NMR spectroscopy

**Figure 2:**
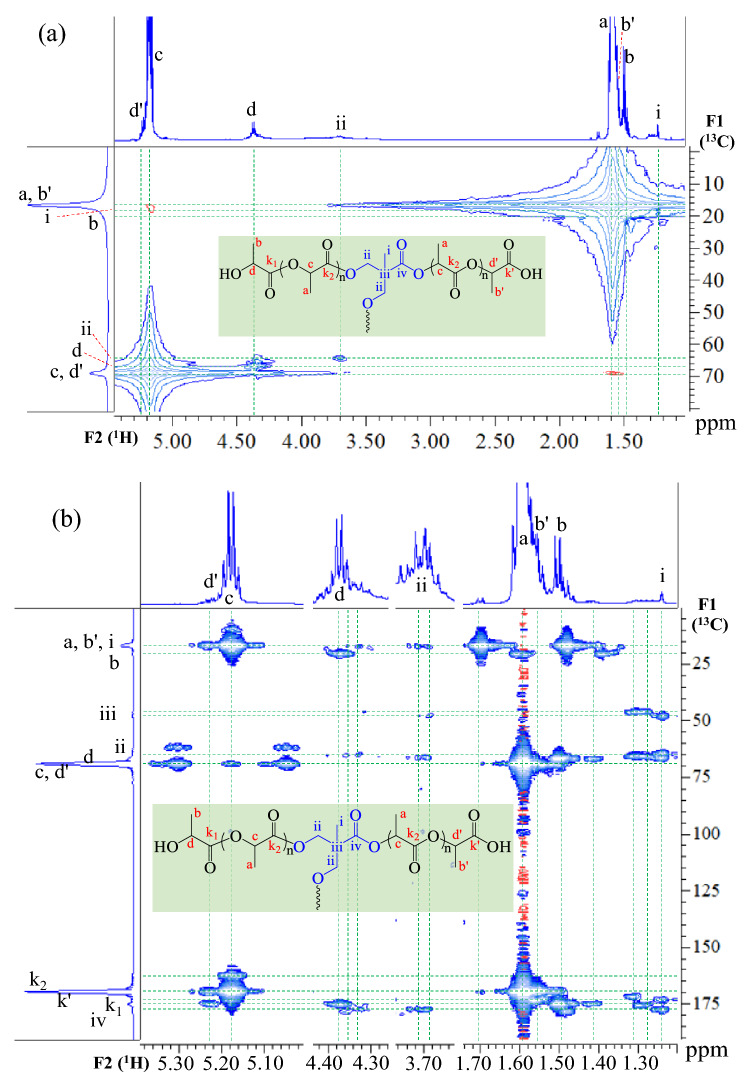
2D-NMR (**a**) HMQC and (**b**) HMBC correlation plots of LD12M.

**Figure 3:**
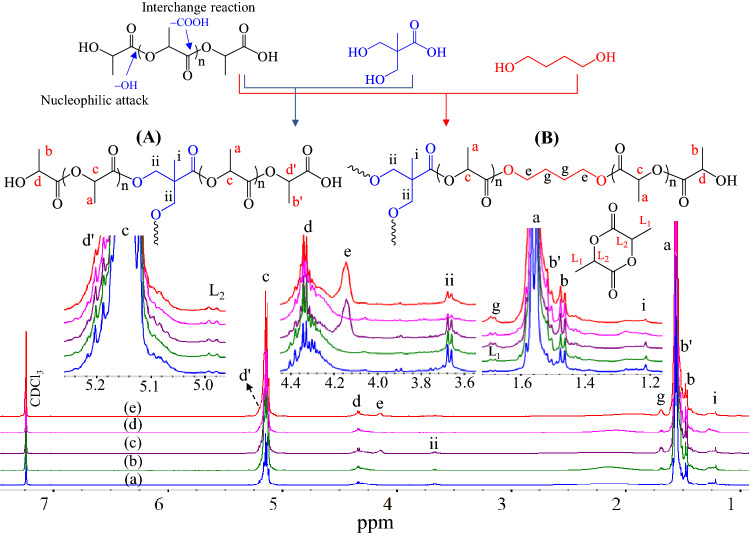
^1^H-NMR spectra and signal assignments of alcohol-acidolyzed PLA products: (**a**) LD6M, (**b**) LD12M, (**c**) LD12B0.2 M, (**d**) LD16M, and (**e**) LD16B0.2 M (500 MHz).

The mechanism of alcoholysis of PLA was discussed in detail in our previous work^33,51^. The acidolysis occurred through the bond interchange reaction between the ester bonds of PLA and the carboxylic group of DMPA^84^. The alcohol-acidolysis mechanisms and chemical structures of the products are illustrated in Figs. 1, 2 and 3. The structural elucidation of the products is conducted using 2D-correlation NMR spectroscopy. An HMQC is employed to examine the direct attachment of ^1^H-^13^C coupling correlation, while HMBC is used to observe long-range correlations of two- and three-bond couplings^85^. The HMQC plot of LD12M is illustrated in Fig. 2a, whose signal assignments are summarized in Supplemental Table S3. The results indicate that the products contain hydroxyl and carboxylic terminals, lactate repeat units, and reacted DMPA core. The signal assignments of three different methyl groups are b(20.40 ppm), b'(16.64 ppm), and a(16.64 ppm) on the F1(^13^C) axis correlated with ^1^H signals of b(1.50 ppm), b'(1.56 ppm), and a(1.59 ppm) on the F2(^1^H) axis. The methine groups of d(66.73 ppm), d'(69.02 ppm), and c(69.02 ppm) on the F1(^13^C) axis correlate with the signals of d(4.37 ppm), d'(5.22 ppm), and c(5.18 ppm), respectively, on the F2(^1^H) axis^86–88^. The core DMPA is reflected by the presence of methyl i(17.70 ppm) and methylene ii(65.00 ppm) on the F1(^13^C) axis, correlated with the signals of i(1.24 ppm) and methylene ii(3.69 ppm) on the F2(^1^H) axis.

The corresponding HMBC plot of LD12M is shown in Fig. 2b, while the information on long-range correlations of two- and three-bond couplings are summarized in Supplemental Table S3. The hydroxyl terminals of lactate are associated with the methyl b(20.40 ppm), methine d(66.73 ppm), and carbonyl k_1_(175.15 ppm) bands on the F1 axis, correlated respectively to the signals (c, d, a), (a, b), and (c, d, a, b) on the F2 axis. The carboxylic terminals of lactate are reflected by the ^13^C bands of methyl b'(16.64 ppm), methine d'(69.02 ppm), and carbonyl k'(169.50 ppm), correlated to the ^1^H signal of (d', c), (c, a, b'), and (d', c, b'), respectively. In addition, the lactate repeat units are represented by the methyl a(16.64 ppm), methine c(69.02 ppm), and carbonyl k_2_(169.62 ppm) signals on the F1(^13^C) axis, correlated to the signals (c, ii, b), (c, a, b), and (d', c , d, a, b', b) on the F2(^1^H) axis^86–88^. The DMPA units covalently connected with lactate sequences exhibit the methyl i(17.70 ppm), iii(47.50 ppm), methylene ii(66.10 ppm), and carbonyl iv(177.50 ppm) signals on the F1 axis, correlated to the signals (ii), (ii, i), (ii, a, i), and (c, ii, a, i) on the F2 axis, respectively.

The information on signal assignments is used in the structural characterization and quantitative analysis of the alcohol-acidolyzed PLA after purification employing ^1^H-NMR spectra, as illustrated in Fig. 3. The use of a 500 MHz ^1^H-NMR spectrometer leads to the shift of all signal positions to a slightly lower frequency than those observed from the 2D-NMR (600 MHz) analysis due to analytical tool resolution. The covalent attachments of lactate sequences and DMPA are defined as structure (A), while those connected to BDO are called structure (B). In these structures, the signals of lactate repeat units were observed at methyl (a ~ 1.56 ppm) and methine (c ~ 5.14 ppm). The traces of hydroxyl terminals of lactate sequences were at b ~ 1.47 and d ~ 4.33 ppm^86^. The carboxyl terminals were reflected by the bands b' ~ 1.51 and d' ~ 5.20 ppm. The presence of DMPA in the core structure was confirmed by the methyl (i ~ 1.21 ppm) and methylene (ii ~ 3.67 ppm) signals. In addition, the α-methylene g ~ 1.67 ppm and β-methylene e ~ 4.14 ppm signals indicate the reaction between PLA and BDO. The lactide cyclic dimer co-product was also detected, reflected by the bands at L_1_ ~ 1.69 and L_2_ ~ 4.99 ppm of the methyl and methine of the lactide ring.

The quantitative analysis results from ^1^H-NMR spectra are summarized in Table 1. The contents of in-chain lactate sequences increased from 85.1 to 90.1 mol% with the increase in the feed PLA: DMPA ratio from 6:1 to 16:1, when only DMPA was employed in the reaction. The corresponding contents of lactate and DMPA core structure slightly decreased from 77.1 to 79.5 mol% and from 5.6 to 4.1 mol%, when BDO and DMPA were introduced. The contents of BDO in the chain structure ranged from 13.3 to 11.7 mol%. These are higher than DMPA, indicating its higher reactivity towards transesterification of PLA compared to DMPA. A small amount of unreacted DMPA was removed during the purification process, as this is soluble in water and acetone. Therefore, the purified products contain only reacted DMPA and BDO units in the core structures conjugated with lactate sequences. Their average molecular weight (${\overline{M} }_{n}$) ranged from 2510 to 4270 g/mol (^1^H-NMR) and 2790 to 4550 g/mol (GPC), with a polydispersity index (PDI) of 2.20–2.55.

**Table 1:** Summary of chemical compositions and molecular weights of alcohol-acidolyzed PLA products.

Sample	^1^H-NMR spectra						GPC results		
	DP*	Structure (A) or (B) compositions			Lactide (mol%)	${\overline{M} }_{n}$ (g/mol)	${\overline{M} }_{n}$ (g/mol)	${\overline{M} }_{w}$ (g/mol)	PDI (${\overline{M} }_{w}$/${\overline{M} }_{n}$)
		polylactate (mol%)	DMPA (mol%)	BDO (mol%)					
LD6M	11.0	85.1	11.6	–	3.3	2,510	2,790	6,150	2.20
LD12M	17.9	88.1	7.3	–	4.6	4,010	4,320	9,550	2.21
LD16M	19.1	90.1	4.9	–	5.0	4,270	4,550	11,520	2.53
LD12B0.2 M	16.1	77.1	5.6	13.3	3.9	3,680	3,670	8,710	2.37
LD16B0.2 M	17.2	79.5	4.1	11.7	4.8	3,910	4,260	10,860	2.55

*DP is the degree of polymerization.

### Chemical structures of alcohol-acidolyzed PLA products by FTIR spectroscopy

**Figure 4:**
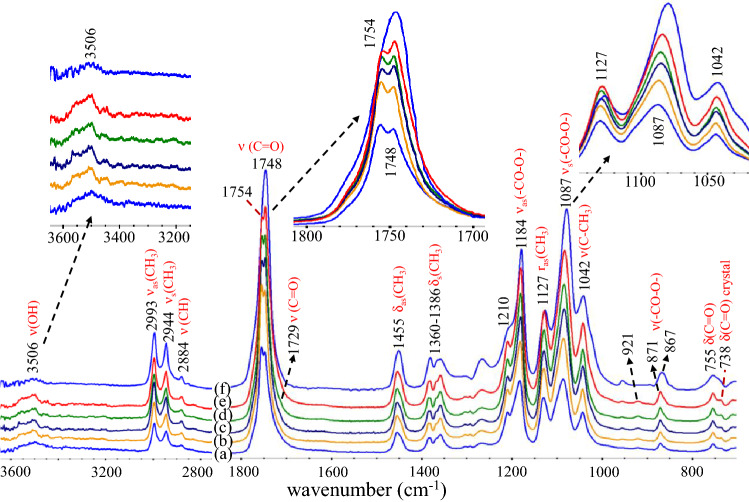
ATR-FTIR spectra of alcohol-acidolyzed PLA products: (**a**) LD6M, (**b**) LD12B0.2 M, (**c**) LD16B0.2 M, (**d**) LD12M, (**e**) LD16M, and (**f**) neat PLA.

The alcohol-acidolysis of PLA by DMPA is examined by FTIR spectroscopy to observe the reaction mechanisms. FTIR spectra of purified products are illustrated in Fig. 4. The bands at 2993, 2944, and 2884 cm^−1^ are associated with the stretching modes of ν_as_(CH_3_), ν_s_(CH_3_), and ν(CH) of PLA domains. The bands of δ_as_(CH_3_), δ_s_(CH_3_), ν_as_(–CO–O-), and ν_s_(–CO–O–) were observed at 1455, 1360–1386, 1184, and 1087 cm^−1^. The ν(OH), ν(C = O), and δ(OH) bands of the carboxylic group of DMPA were detected at 3361, 1688, and 1308 cm^−1^. The ν(OH) and δ(OH) bands of hydroxyls of DMPA are located at 3221, 1232, and 938 cm^−1^. After the reaction, the O–H bands of DMPA from carboxylic and hydroxyl groups disappeared, reflecting their role in the transesterification of PLA. This indicates the alcoholysis and acidolysis of PLA by DMPA, in which the ester bonds cleave to shorter lactate sequences. An intensity ratio of the 1087/1127 cm^−1^ band indicates the relative chain length of the products, in which a higher value reflects a longer lactate sequence. The acidolysis produces carboxylic terminals for the lactate sequences, reflected by the disappearance of DMPA’s carboxylic acid band at 1688 cm^−1^ and an increase in the intensity of the lactate’s carboxylic at 1729 cm^−1^. As all alcohol-acidolyzed products possess medium molecular weights, the content of hydroxyl terminals is relatively low. These are largely isolated, leading to a lower degree of hydrogen bonding, reflected by a weak ν(OH) stretching band centered at a higher frequency of 3506 cm^−1^. The band overlaps with the C = O overtone modes at 3545 and 3506 cm^−1^^89–93^, due to their intense fundamental mode vibration.

**Figure 5:**
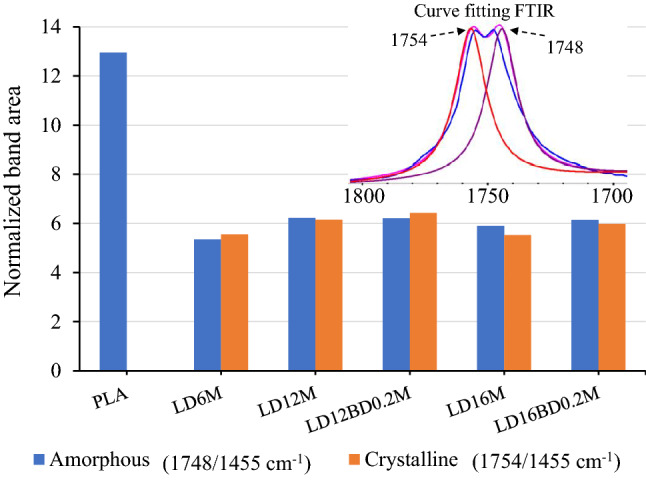
Normalize band areas of the amorphous (1748/1455 cm^-1^) and crystalline (1754/1455 cm^−1^) C = O characteristics of lactate sequences obtained from FTIR curve fitting analysis.

Band splitting of the C = O modes was observed. The 1754 cm^-1^ band is crystalline, while the 1748 cm^−1^ mode is associated with the amorphous characteristic^89–91^. This also agrees with the splitting of the 1382 and 1386 cm^−1^ bands, corresponding to amorphous and crystalline domains^94–96^. The characteristic bands of the crystalline domains were also observed at 1210, 921, 871, and 738 cm^−1^^97,98^. The amorphous/crystalline compositions can be quantitatively determined, as illustrated in Fig. 5. A curve fitting process is applied to determine the areas of the 1748 and 1754 cm^−1^ bands after being normalized to a reference band at 1455 cm^−1^. Neat PLA exhibited a completely amorphous nature, while the alcohol-acidolyzed PLA products showed semi-crystalline structures due to the induction by hydroxyl groups.

### Crystalline structures of alcohol-acidolyzed PLA products

**Figure 6:**
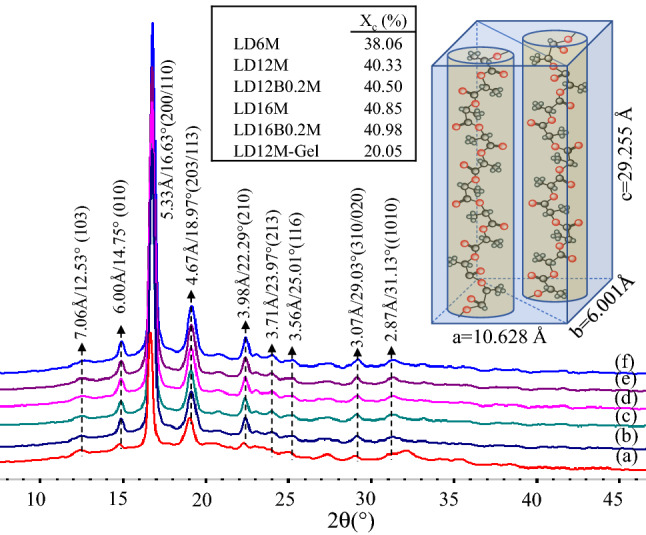
XRD spectra of (**a**) LD12M gel in acetone, (**b**) LD6M, (**c**) LD12M, (**d**) LD12B0.2 M, (**e**) LD16M, and (**f**) LD16B0.2 M, and proposed crystalline structures.

The crystalline structures and crystallinity of alcohol-acidolyzed PLA products are examined by X-ray diffraction (XRD) spectroscopy. XRD traces of purified products are compared in Fig. 6. All products show high crystalline contents, reflected by the sharp and intense crystalline peaks and high relative areas of crystalline peaks per amorphous domains. Four crystal characteristics of PLA are revealed as α (α'), β, γ, and δ^99–105^. All purified products exhibit α-crystal structure, reflected by uniform crystalline peaks attributed to the distorted 10_3_ helical chains in the orthorhombic (pseudo-orthorhombic) unit cell^101,106–108^. The sharp diffraction peaks at 2θ of 14.75(010), 16.63(200), and 18.97°(203) with the corresponding *d*-spacing of 6.00, 5.33, and 4.67 Å were observed for all samples. Relatively weaker peaks at 2θ of 12.53, 22.29, 23.97, 25.01, 29.03, and 31.13° indicate the intense characteristics of the α-crystals form^106,109–111^. The products’ crystalline content (*Xc* %) was 41%. The orthorhombic unit cell distribution of the crystalline domain was calculated from Bragge’s equation: (*1*/*d*_*hkl*_)^2^ = (*h*/a)^2^ + (*k*/b)^2^ + (*l*/c)^2^, where the (*hkl*) planes are ascribed to (010), (200), and (203). The unit cell possesses the crystal dimension of a = 1.0628, b = 0.6001, and c = 2.9255 nm. These differ slightly from neat PLA (a = 1.07, b = 0.595, and c = 2.78 nm)^107,108,112^. The larger unit cell observed in these alcohol-acidolyzed PLA structures is likely due to the presence of DMPA and BDO in the chain structure, affecting the chain folding distance and the degree of chain symmetry. The inclusion of DMPA and BDO in the structure of medium-size lactate oligomers, however, does not inhibit the α-crystal formation. Enlargement in the crystalline size of the products is observed.

Interestingly, when the samples were dissolved in a minimum amount of acetone at 40–50 °C in the purification process before precipitation, it was noticed that the polymer/acetone solution became gel when the mixture was cooled down to room temperature overnight. Its crystalline structure is therefore examined. After the gelation of LD12M in acetone, the crystalline content decreased to 20%, while the α-crystal structure changed to disordered α'-conformation. This is reflected by a shift of the characteristic peaks to 12.37, 14.63, 16.57, 18.93, and 22.27°, indicating a slight increase in the *d*-spacing values^110,113^. The orthorhombic cell dimension of a = 1.0692, b = 0.6050, and c = 2.9159 nm was observed. The distance of the two 10_3_ helical chains significantly increased from a = 1.0628 to 1.0692 nm. Also, the diameter of each 10_3_ chains increased from b = 0.6001 to 0.6050 nm, whereas the length of helical chains decreased from c = 2.9255 to 2.9159 nm. The results indicate that the gelation deforms the chain packing structure, as acetone molecules penetrate the polymer's structures by forming hydrogen bonding with methyl side groups or methine of the lactate repeat units.

### Thermal properties of alcohol-acidolyzed PLA products 

**Figure 7:**
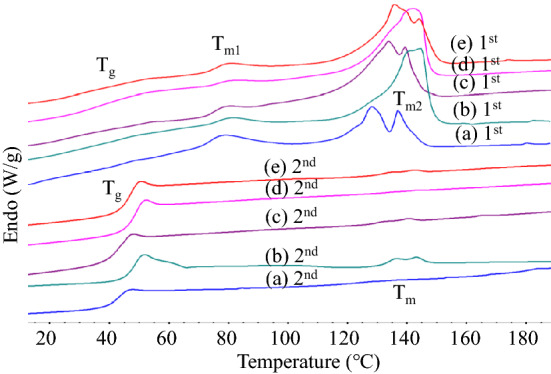
DSC thermograms from the 1st and 2nd heating cycles of alcohol-acidolyzed PLA: (**a**) LD6M, (**b**) LD12M, (**c**) LD12B0.2 M, (**d**) LD16M, (**e**) LD16B0.2 M.

The thermal properties of alcohol-acidolyzed PLA products are examined by DSC, whose thermograms are shown in Fig. 7 and summarized in Table 2. The transesterification of PLA chains by DMPA and BDO to medium-sized products leads to a decrease in its glass transition (T_g_) and melting temperature (T_m_). The T_g_ value observed from the 1^st^ heating thermograms increased from 32.2 to 37.5 °C, when the PLA/DMPA feed ratio increased from 6:1 to 16:1. When a small amount of BDO was introduced along with DMPA, the T_g_ values slightly dropped, due to the increase in the degree of transesterification, leading to shorter lactate sequences. This is a result of the higher reactivity of BDO compared to DMPA. Also, the linear structure of BDO, without a side chain, compared to DMPA, is perhaps responsible for the lowering of the T_g_ value. Two melting characteristics were observed. The melting peaks at a higher temperature range (129.3–141.8 °C), T_m2_, are slightly lower than neat PLA, indicating the characteristic of lactate domains longer than the critical folding period of PLA crystal lamellae. The value decreased with the DMPA feed content, reflecting shorter sequences of the lactate blocks in the structure. The melting characteristics (T_m1_) at a lower range of 77.7–83.3 °C is likely due to the domains of DMPA connected to the lactate sequences in branched structures. It is noted that the ΔH_m_ (J/g) values were calculated from a combination of endotherms T_m1_ to T_m2_. The crystalline content *X*_*c*_(%) was calculated using the equation of *ΔH*_*m*_ × 100*/ΔH*°, where *ΔH*° of 93 J/g was employed^114,115^. The products had crystalline contents in a range of 38–40% when the reaction of PLA with DMPA was employed with or without adding a small amount of BDO. The 2nd heating scan thermograms showed more distinct glass transition behavior but much weaker melting characteristic, likely due to a relatively faster DSC cooling rate than the materials’ melt crystallization rate. The T_g_ value decreased from 47.7 to 42.3 °C when the feed PLA/DMPA ratio varied from 16:1 to 6:1. A small amount of BDO along with DMPA led to a further reduction in the T_g_ value of approximately 2–4 °C. These confirm the higher degree of chain scission and shorter chain lengths.

**Table 2:** Summary of thermal properties of various alcohol-acidolyzed PLA products, derived from the 1st to 2nd heating scan DSC thermograms.

Sample	1st heating				2nd heating			Crystallinity* X_*c*_ (%)
	T_g_ (°C)	T_m1_ (°C)	T_m2_ (°C)	ΔH_m_ (J/g)	T_g_ (°C)	T_m_ (°C)	ΔH_m_ (J/g)	
LD6M	32.2	77.7	131.8	35.0	42.3	132.5	0.56	37.7
LD12M	36.4	80.3	141.8	35.6	47.5	140.1	1.99	38.3
LD12B0.2 M	33.6	78.3	135.9	35.7	43.0	136.1	0.51	38.4
LD16M	37.5	80.0	141.7	35.7	47.7	140.0	0.50	38.4
LD16B0.2 M	36.3	79.3	139.2	36.8	45.9	138.7	0.52	39.6

*Calculated from the 1st heating scan thermogram,* X*_*c*_ (%) = (*ΔH*_*m*_-*ΔH*_*c*_) × 100/*ΔH*°, where *ΔH*° (93 J/g).

### PLA-based PUD derived from alcohol-acidolyzed PLA products

**Figure 8:**
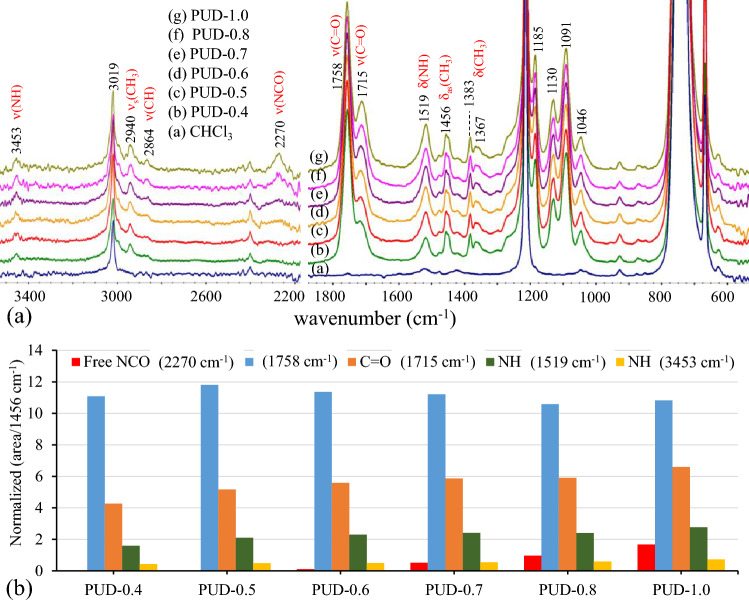
(**a**) ATR-FTIR spectra and (**b**) normalized band areas of PUD/CHCl_3_ solutions obtained from different feed ratios of LD12M and HDI.

PLA-based polyurethanes (PUD) are synthesized from the alcohol-acidolyzed PLA products by reacting with HDI. FTIR spectra of PUD synthesized from LD12M are illustrated in Fig. 8a. Characteristic bands of lactate sequences similar to those of the starting alcohol-acidolyzed PLA were observed. The newly-formed urethane bonds are confirmed by the bands at 3453, 1715, and 1519 cm^−1^, associated with the ν(NH), ν(C = O), and δ(NH) modes, respectively. The NCO band at 2270 cm^−1^ was observed in PUD-0.6 to PUD-1.0 samples, reflecting an excess unreacted HDI in the system. In contrast, the band was not detected in PUD-0.4 and PUD-0.5, indicating an equimolar of the hydroxyl terminals in LD12M and NCO groups of HDI. Additionally, the normalized band areas of free NCO groups and urethane bond formation are summarized in Fig. 8b, in which the δ_as_(CH_3_) band at 1456 cm^−1^ was used as a reference band. The results indicate an increase in the urethane bond content as a function of the HDI amount. These are reflected by the rise of the normalized band areas of the NH (3453 cm^−1^), C = O (1715 cm^−1^), and NH (1519 cm^−1^) vibrational modes. The normalized area of the NCO(2270 cm^−1^) band indicates the content of unreacted HDI. The value increased from PUD-0.6 to PUD-1.0.

### Particle size and Zeta potential of alcohol-acidolyzed PLA emulsions

**Figure 9:**
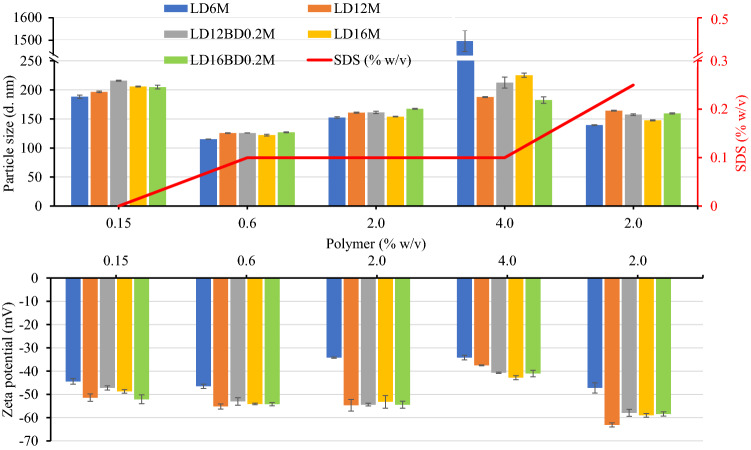
Particle size and Zeta potential of emulsions of alcohol-acidolyzed PLA products fabricated with and without SDS surfactant.

The alcohol-acidolyzed PLA products obtained from different feed conditions and the corresponding PUD/CHCl_3_ solutions are employed in fabricating mico-/nanoparticle emulsions with and without external surfactants, *i.e.*, SDS. The particle size and zeta potential of the emulsions from different samples as a function of total solid content (TSC) and SDS concentrations are examined by Zetasizer with polystyrene reference. In the system without SDS surfactant, the TSC was fixed at 0.15 w/v% to the aqueous solution. It was observed that the particles agglomerated and precipitated at the bottom of the container, while some smaller particles dispersed in the aqueous medium. The remaining dispersion showed an average size of 188 nm with a broad PDI value (> 0.267), as shown in Fig. 9 and tabulated in Supplemental Table S4. The Zeta potential values of greater than −44 mV reflect that the particles have excellent stability in an aqueous medium. This high negative charge value is possibly generated from the deprotonation of the carboxylic acid terminals in their structures. This confirms that the materials can be used as a self-stabilizable polymer matrix in the emulsion formation at a low TSC value. The introduction of SDS as a surfactant significantly improved the particle stability with higher Zeta potential, smaller size (108–139 nm), and low PDI value, when a TSC of 0.6 w/v% with 0.1w/v% SDS content was applied. The particle size slightly increased with insignificant changes in Zeta potential when the TSC was increased to 2 w/v% at SDS contents of 0.1–0.25 w/v%. However, when the TSC was further increased to 4 w/v%, the particle size sharply increased to 200 nm with a dropped in the Zeta potential value. This indicates the particle’s instability and agglomeration.

The effects of alkaline and acidic environments on the particle’s stability are examined on LD12M by fixing its TSC value and adjusting the pH of the aqueous medium with NaOH or HCl, as summarized in Table 3. The emulsion prepared using SDS as a surfactant possesses the highest Zeta potential at −55.2 mV, and a small average particle size of 125.8 ± 0.5 nm, indicating its high performance in stabilizing the particles. Interestingly, without SDS, the emulsion fabricated using basic pH exhibited a slightly lower Zeta potential, with a slightly larger size when 0.01 M NaOH was employed. The average size increased, and the negative charge value decreased as the NaOH concentration decreased from 0.01 to 0.001 M. This confirms that the alkaline medium induces the deprotonation of carboxylic acid terminals, generating negative carboxylates at the particle’s surfaces. The Zeta potential values of the emulsions with SDS and SDS/NaOH (0.01 M) were significantly similar but slightly higher than that in NaOH. The results firmly indicate that the deprotonation of the carboxylic acid end-groups is effective in stabilizing the emulsion system. In contrast, a stable emulsion cannot be formed in acidic environments when an HCl solution is employed at any concentration. Agglomeration and precipitation of the polymeric particles were observed. The combination of SDS surfactant with alkaline or acidic environments insignificantly affected the particle size and zeta potential of the emulsions, whose size of 122–127 nm and Zeta potential values of −50 to −59 mV were obtained (Table 3). The results imply that SDS surfactant plays a more important role in the particle’s stability and size, due to its highly negatively charged nature.

**Table 3:** Summary of particle size and Zeta potential of emulsions of alcohol-acidolyzed PLA in alkaline and acid environments.

Sample	Composition				Emulsion properties		
	Polymer % w/v	SDS (% w/v)	NaOH (M)	HCl (M)	Particle size (nm)	PDI	Zeta potential (mV)
LD12M	0.6	0.1	–	–	126 ± 1	0.12 ± 0.02	−55.2 ± 1.1
LD12M	0.6	–	0.01	–	138 ± 1	0.17 ± 0.01	−55.2 ± 1.6
LD12M	0.6	0.1	0.01	–	123 ± 1	0.19 ± 0.01	−51.1 ± 0.5
LD12M	0.6	–	0.001	–	149 ± 1	0.12 ± 0.01	−49.7 ± 0.9
LD12M	0.6	0.1	0.001	–	122 ± 1	0.14 ± 0.01	−59.1 ± 2.0
LD12M	0.6	0.1	0.0001	–	126 ± 1	0.14 ± 0.01	−50.2 ± 1.1
LD12M	0.6	0.1	–	0.01	122 ± 1	0.17 ± 0.01	−52.6 ± 1.3
LD12M	0.6	0.1	–	0.001	127 ± 1	0.16 ± 0.01	−49.6 ± 0.7

### Particle size and Zeta potential analysis of PLA-based PUD emulsions

The effects of SDS surfactant and alkaline (NaOH) medium on the particle size and Zeta potential of the emulsions prepared from the synthesized PLA-based PUD are summarized in Table 4. PUD-0.4 and PUD-0.5 were chosen as these are derived from equimolar reagents, *i.e.*, unreacted NCO groups of HDI were not detected. In the emulsions containing SDS surfactant, the particle size distribution was small as 124 nm with a Zeta potential of −49.0 to −54.9 mV. These emulsions were obtained with narrow PDI values, indicating monodispersed particle size. However, the emulsion of PUD-0.5 in an alkaline NaOH (0.001 M) medium increased the particle size to 144 nm, with a similar Zeta potential to the SDS system. This implies deprotonation of the remaining carboxylic acid of PUD-0.5, generating negative charges that help to stabilize the polymer particles.

**Table 4:** Summary of particle size and Zeta potential of PLA-based PUD emulsions in the presence of SDS surfactant and alkaline environment.

Sample	Emulsion properties					
	Polymer % w/v	SDS (% w/v)	NaOH (M)	Particle size (nm)	PDI	Zeta potential (mV)
PUD-0.4	0.51	0.1	–	125 ± 1	0.16 ± 0.01	−48.5 ± 0.6
PUD-0.5	0.53	0.1	–	124 ± 1	0.13 ± 0.01	−54.9 ± 0.2
PUD-0.5	0.53	–	0.001	144 ± 1	0.13 ± 0.01	−54.8 ± 0.9

### Morphology of alcohol-acidolyzed PLA and PLA-based PUD particles

**Figure 10:**
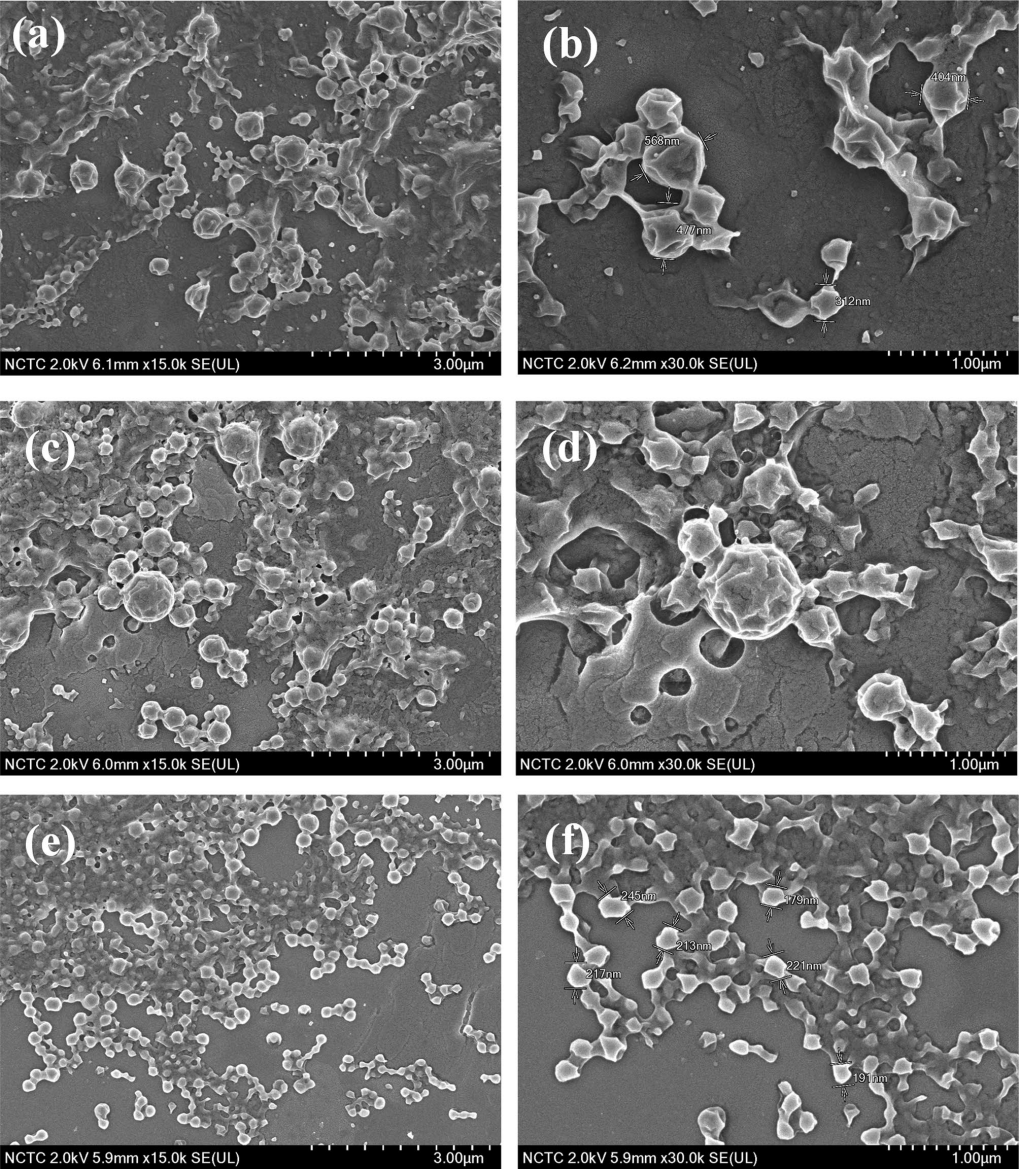
SEM images of alcohol-acidolyzed PLA particles obtained from different emulsion preparation conditions: (**a**, **b**) LD12M(2%)-SDS(0.1%), (**c**, **d**) LD12M(2%)-SDS(0.1%)-NaOH(0.001 M), and (**e**, **f**) LD12M(2%)-SDS(0.1%)-HCl(0.001 M).

The morphology of alcohol-acidolyzed PLA particles obtained from different emulsion preparation conditions was examined under SEM analysis. The emulsions were cast on a thin glass slide and dried at room temperature. SEM images of LD12M(2%)-SDS(0.1%) without NaOH are shown in Fig. 10a,b. Particles with defective spherical shapes and rough surfaces were observed with non-uniform size distribution. Each particle consists of crystallite lamellae, forming a shape. This is likely because the materials have medium-sized chains and a high content of hydroxyl and carboxyl terminals which may induce and enhance the formation of separate crystalline lamellae. The stability of the oligomer droplets during the emulsification process due to the presence of SDS enables the crystal flakes to stick together and form spherical particles. When an alkali medium is employed with SDS, as shown in Fig. 10c,d, particles with a more regular spherical shape were obtained due to the combined stabilization effects of the surfactant and carboxylate groups on the surface droplets. In contrast, when HCl (0.001 M) was used, as shown in Fig. 10e,f, separate crystallite flakes of small-sized lamellae were formed. This is because of the low stability of the emulsion droplets, which retard the formation of spherical structures in the acidic environment.

The morphology of the corresponding PLA-based PUDs particles prepared from different emulsion preparation conditions is also examined, whose SEM images are compared in Supplemental Figure S1. Similar spherical particles consisting of crystallite lamellae were also observed. However, the particles tended to agglomerate and merged together to form a film-like surface. This reflects the larger chain structures and higher adhesion between the polyurethane chains due to their strong hydrogen bonding interaction.

### Lavender oil-loaded PLA-based PUD nanoparticles 

Lavender oil (LO)-loaded PUD nanoparticles were prepared via an O/W emulsion technique. A mixture of LO and PUD in chloroform was prepared and added into an aqueous phase. The mixture was separated into two phases. During a high-shear treatment, the oil-dispersed phase breaks down, resulting in droplet formation. The solubility of PUD was disturbed by the water, which is a good solvent for the hydrophilic copolymer. After solvent evaporation, LO was encapsulated in the shell of PUD nanoparticles. Besides electrostatic stabilization, the interaction between the hydrophilic PUD segments and LO molecules is responsible for the repulsion of the particles.

The hydrodynamic size, PDI, and Zeta potential of the prepared nanoparticles are summarized in Table 5. The particle size of PUD@LO nanoparticles significantly increased with an increasing amount of PUD. At PUD: LO ratios of 1.25:1 and 2.50:1, *i.e.*, PUD1.25@LO and PUD2.5@LO, the hydrodynamic size of nanoparticles were 318 and 342 nm, respectively, with a narrow size distribution (PDI = 0.28). When the ratio was increased to 3.75:1 (PUD3.75@LO), large nanoparticles (524 nm) with a broad size distribution (PDI = 0.46) were produced. A further increase in the PUD content beyond this condition led to the precipitation of solid PUD at the bottom of the reactor. This phenomenon can be explained based on the viscosity of the dispersed phase. The increase in polymer content leads to an increase in the viscous forces resisting droplet breakdown by mechanical energy. As a result, larger-sized droplets are formed, generating bigger nanoparticle diameters^116^.

**Table 5:** Summary of particle size, PDI, Zeta potential, %EE, and %LC of PUD@LO samples from different emulsion preparation conditions.

Samples	Particle size (nm)	PDI	Zeta potential (mV)	%EE	%LC
PUD1.25@LO	318 ± 9	0.28	−66.7 ± 2.0	80 ± 0.1	64 ± 0.1
PUD2.50@LO	342 ± 4	0.28	−64.7 ± 1.4	84 ± 0.1	34 ± 0.1
PUD3.75@LO	524 ± 10	0.46	−65.5 ± 1.2	88 ± 0.1	24 ± 0.0

Moreover, a correlation between the PUD content and the oil encapsulation efficiency was observed. The %EE value increased with the polymer content. The encapsulation of LO in PUD nanoparticles is achieved by direct encapsulation during the particle’s formation step. Both LO and PUD are well dissolved in the chloroform oil phase. The swollen PUD matrix may adsorb LO due to their high compatibility. After solvent evaporation, hence, a high volume of the hydrophobic segment PUD could accumulate a high amount of LO. This emphasizes the oil-polymer association, which is confirmed by a drastic decrease in the %LC value. All prepared PUD@LO nanoparticles exhibited high colloidal stability in the alkaline aqueous (0.001 M NaOH) without using any surfactant, reflecting a high self-stabilizability of the deprotonated carboxylate groups at a pH > 4.2 (pKa of carboxylic acid)^117^. The results agree well with their negative Zeta potential values at pH 8, which are higher than -64 mV, as summarized in Table 5.

**Figure 11:**
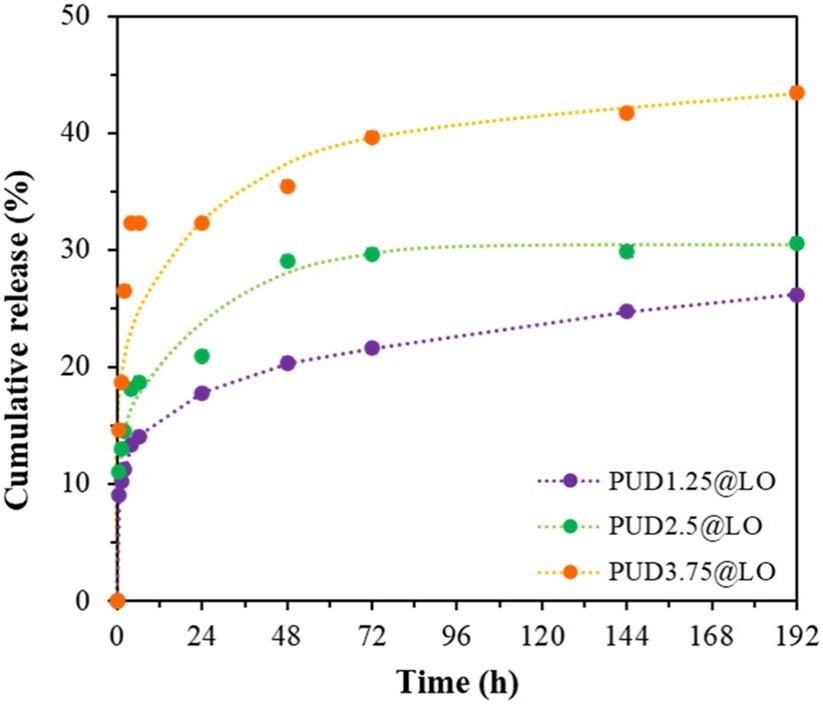
Release profiles of LO from PUD@LO nanoparticles at 37 °C in PBS (pH 8.5).

The in vitro release behavior is critical to the safety, efficacy, and quality of nanoparticle-based drug delivery systems. As PUD@LO cannot form a stable cast film, the solid particles are employed by embedding them in a film-forming PVA substrate. The material's release behavior due to LO diffusion through the polymeric matrix at pH 8.5, which is the pH of wound exudate, was examined to assess its potential use in wound healing and cell scaffold applications^83^. The release profiles of LO within 192 h (8 days) are shown in Fig. 11. All PUD@LO nanoparticles displayed a similar release profile. An initial high release rate was observed (burst effect) as parts of the hydrophobic LO may be trapped on the surface of the PUD matrix during the preparation process. In the subsequent release phase, the LO release is controlled by its diffusion through the polymeric PUD shell^118^. It is noted that particle size significantly affects the rate of LO release. As the nanoparticle size increased, the percentage of LO release increased. This is likely due to the higher free surface area of the larger particles embedded on the PVA film substrate, leading to an enhanced diffusion rate of LO through the PUD shell structure. Insights into this release behavior will be investigated in a separate communication.

### Cytotoxicity of PUD@LO nanoparticles

**Figure 12:**
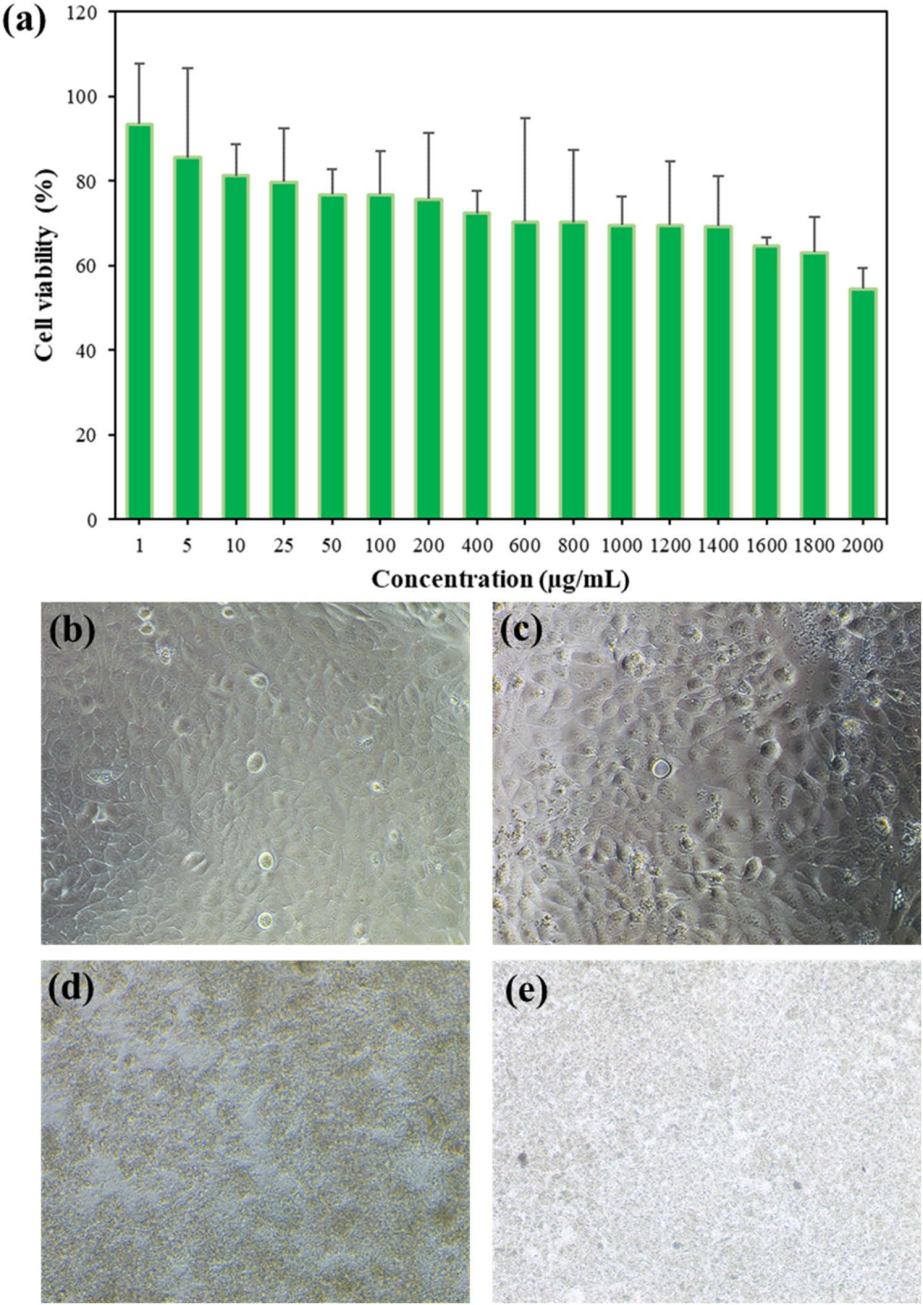
(**a**) Cell viability of HaCaT after incubation with PUD@LO nanoparticles at various concentrations: (**b**) 0 (control), (**c**) 50, (**d**) 1000, and (**e**) 1800 µg/mL.

The cytotoxicity assay is used to examine the biocompatibility of the prepared PUD@LO nanoparticles. The toxicity of PUD3.75@LO was studied using an MTT assay. After incubation, as shown in Fig. 12a, the viability of HaCaT cells slightly decreased with the nanoparticle concentration. The value was still higher than 70% at a concentration as high as 1800 µg/mL. Moreover, to confirm the biocompatibility of PUD@LO nanoparticles, the cell growth morphology was examined using a conventional light microscope. The images were captured from each sample consisting of HaCaT cells incubating with PUD@LO nanoparticles at different concentrations. At low concentrations of PUD@LO nanoparticles (50 µg/mL) in Fig. 12c, no significant difference in cell confluency and morphology was observed compared to the control in Fig. 12b. At high concentrations of 1000–1800 µg/mL in Figs. 12d,e, however, the morphology of cells was not clearly detected due to the high amount of nanoparticles. The IC_50_ value (concentrations of the substances that exhibited 50% of cell viability) of PUD@LO nanoparticles were higher than 2000 µg/mL, reflecting their high compatibility with the cells. The as-prepared PUD@LO nanoparticles have a potential for further in vivo use.

## Conclusions

An alcohol-acidolysis reaction was successfully employed to convert PLA resin into lactate oligomers containing hydroxyl and carboxylic acid terminals by utilizing DMPA and BDO. The microwave-assisted reaction was conveniently conducted at 220–240 °C and pressure lower than 100 psi. PLA resin was completely converted, with a product yield as high as 93%. The purified products were characterized by FTIR, 2D-NMR, ^1^H-NMR, GPC, DSC, and XRD spectroscopy. The chemical structures, molecular weights, and particle formability of the oligolactate products strongly depended on the PLA: DMPA feed ratio and the incorporation of BDO. The product from a ratio of 12:1, which possessed optimum size and structures, was used to synthesize PLA-based polyurethane (PUD) by reacting with HDI at an equimolar ratio. The resulting PUD was employed in encapsulating lavender essential oil (LO). Without using any surfactant, stable LO-loaded nanoparticles were prepared with a Zeta potential value of greater than -60 mV due to the copolymer’s self-stabilizability from its carboxylate groups. At the polymer: LO feed ratios of 1.25:1 to 3.75: 1, a variation in the physicochemical properties of the resulting nanoparticles was observed, e.g., hydrodynamic size (300–500 nm) and encapsulation efficiency (80–88%). The in vitro release of LO from the PUD@LO nanoparticles was investigated. An initial high release rate was observed (burst effect) as parts of the hydrophobic LO may be trapped on the particle’s surface, followed by the diffusion of encapsulated LO through the polymeric PUD shell. The particle size significantly affected the rate of LO release. As the nanoparticle size increased, the percentage of LO release increased. The cytotoxicity of PUD@LO nanoparticles was examined using HaCaT cells. An IC_50_ value higher than 2000 µg/mL was observed, reflecting their high compatibility. The results confirm that the products have high potential as drug encapsulation templates, hydrogel, ionic polymer, and functionalized PUs in biomedical applications.

## Supplementary Information

Supplementary Information.

## Data Availability

The datasets generated and/or analyzed during the current study are available in the “Materials Cloud” repository, https://doi.org/10.24435/materialscloud:3k-98.
