# Supplementary material for: Sizing down and functionalizing polylactide (PLA) resin for synthesis of PLA-based polyurethanes for use in biomedical applications
Source: Sci Rep. 2023 Feb 9;13:2284. doi: 10.1038/s41598-023-29496-x (PMC9911729; doi:10.1038/s41598-023-29496-x)
Supplement: Supplementary file 1 — Supplementary Information. [file 41598_2023_29496_MOESM1_ESM.docx]

**Supplemental Data**

**Sizing Down and Functionalizing Polylactide (PLA) Resin for Synthesis of PLA-based Polyurethanes for Use in Biomedical Applications**

Bunthoeun Nim^1^, Sosna Rahayu^1^, Kamonchanok Thananukul^1^, Chorney Eang^1^,

Mantana Opaprakasit^2^, Atitsa Petchsuk^3^, Chariya Kaewsaneha^1^, Duangporn Polpanich^4^,

Pakorn Opaprakasit^1*^

*^1^ School of Integrated Science and Innovation, Sirindhorn International Institute of Technology (SIIT), Thammasat University, Pathum Thani, 12121, Thailand.*

*^2^ Department of Materials Science, Faculty of Science, Chulalongkorn University,*

*Bangkok, 10330, Thailand.*

*^3^ National Metal and Materials Technology Center (MTEC), National Science and Technology Development Agency (NSTDA), Pathum Thani, 12120, Thailand.*

*^4^ National Nanotechnology Center (NANOTEC), National Science and Technology Development Agency (NSTDA), Thailand Science Park, Pathum Thani,12120, Thailand*

**E-mail: pakorn@siit.tu.ac.th*

**Table S1:** Summary of alcohol-acidolysis conditions of PLA resin in a microwave reactor.

| Sample |  | PLA/DMPA/BDO ratio | | Conditions  10 min; <100 psi | Product yield  (%) |
| --- | --- | --- | --- | --- | --- |
|  |  | by wt. | by mol |  |  |
| LD6M |  | 6:1:0 | 11.2:1:0 | 220 ℃ | 82.6 |
| LD12M |  | 12:1:0 | 22.4:1:0 | 220 ℃ | 89.7 |
| LD16M |  | 16:1:0 | 29.8:1:0 | 240 ℃ | 91.2 |
| LD12BD0.2M |  | 12:0.8:0.2 | 27.9:1:0.4 | 220 ℃ | 88.5 |
| LD16BD0.2M |  | 16:0.8:0.2 | 37.3:1:0.4 | 220 ℃ | 93.2 |

**Table S2:** The synthesis conditions of PLA-based PUD from alcohol-acidolyzed PLA products.

| Sample | LD12M | | HDI | | Hydroxyl: HDI  molar ratio |
| --- | --- | --- | --- | --- | --- |
|  | (g) | Mole * | mL | Mole |  |
| PUD-0.4 | 3.0 | 0.000748 | 0.40 | 0.00250 | 1 : 3.3 |
| PUD-0.5 | 3.0 | 0.000748 | 0.50 | 0.00312 | 1 : 4.2 |
| PUD-0.6 | 3.0 | 0.000748 | 0.60 | 0.00375 | 1 : 5.0 |
| PUD-0.7 | 3.0 | 0.000748 | 0.70 | 0.00437 | 1 : 5.8 |
| PUD-0.8 | 3.0 | 0.000748 | 0.80 | 0.00499 | 1 : 6.7 |
| PUD-1.0 | 3.0 | 0.000748 | 1.00 | 0.00624 | 1 : 8.3 |

## *Calculated from ^1^H-NMR spectra

**Table S3:** Summary of signal correlations of alcohol-acidolyzed PLA products obtained from HMQC and HMBC 2D-NMR spectroscopy.

| **HMQC: LD12M** | | **HMBC: LD12M** | |
| --- | --- | --- | --- |
| F1(^13^C ppm) | F2(^1^H ppm) | F1(^13^C ppm) | F2(^1^H ppm) |
| i(17.70) | i(1.24) | i(17.70) | ii |
| b(20.40) | b(1.50) | b(20.40) | c, d, a |
| b'(16.64) | b'(1.56) | b'(16.64) | d', c |
| a(16.64) | a(1.59) | a(16.64) | c, ii, b |
| ii(65.00) | ii(3.69) | ii(66.10) | ii, a, i |
| d(66.73) | d(4.37) | d(66.73) | a, b |
| c(69.02) | c(5.18) | c(69.02) | c, a, b |
| d'(69.02) | d'(5.22) | d'(69.02) | c, a, b' |
|  |  | iii(47.50) | ii, i |
|  |  | k2(169.62) | d', c , d, a, b', b, |
|  |  | k'(169.50) | d', c, b' |
|  |  | k1(175.15) | c, d, a, b |
|  |  | iv(177.50) | c, ii, a, i |

**Table S4:** Summary of particle size and Zeta potential of emulsions of alcohol-acidolyzed PLA products.

| Sample | Aqueous phase | | Particle size (nm) | | Zeta potential  (mV) |
| --- | --- | --- | --- | --- | --- |
|  | Polymer (% w/v) | SDS (% w/v) | Average | PDI |  |
|  | 0.15 | 0 | 188.3 ± 2.8 | 0.307 ± 0.036 | -44.4 ± 1.2 |
|  | 0.6 | 0.1 | 115.1 ± 0.3 | 0.208 ± 0.003 | -46.5 ± 0.9 |
| LD6M | 2.0 | 0.1 | 152.5 ± 1.2 | 0.237 ± 0.004 | -34.2 ± 0.3 |
|  | 4.0 | 0.1 | 1494 ± 46.8 | 1.000 ± 0.001 | -34.2 ± 1.0 |
|  | 2.0 | 0.25 | 139.4 ± 0.7 | 0.194 ± 0.012 | -47.2 ± 2.2 |
|  | 0.15 | 0 | 196.6 ± 1.4 | 0.267 ± 0.006 | -51.4 ± 1.6 |
|  | 0.6 | 0.1 | 125.8 ± 0.5 | 0.119 ± 0.021 | -55.2 ± 1.1 |
| LD12M | 2.0 | 0.1 | 160.7 ± 1.1 | 0.148 ± 0.030 | -54.7 ± 2.5 |
|  | 4.0 | 0.1 | 187.7 ± 0.8 | 0.422 ± 0.015 | -37.5 ± 0.2 |
|  | 2.0 | 0.25 | 164.3 ± 0.7 | 0.204 ± 0.006 | -63.1 ± 0.9 |
|  | 0.15 | 0 | 216.0 ± 0.9 | 0.384 ± 0.002 | -47.2 ± 0.9 |
|  | 0.6 | 0.1 | 125.8 ± 0.2 | 0.119 ± 0.010 | -53.0 ± 1.6 |
| LD12B0.2M | 2.0 | 0.1 | 161.2 ± 2.1 | 0.161 v 0.019 | -54.4 ± 0.6 |
|  | 4.0 | 0.1 | 212.5 ± 9.5 | 0.400 ± 0.015 | -40.7 ± 0.3 |
|  | 2.0 | 0.25 | 157.4 ± 1.4 | 0.192 ± 0.008 | -57.9 ± 1.5 |
|  | 0.15 | 0 | 205.8 ± 0.8 | 0.383 ± 0.031 | -48.7 ± 0.8 |
|  | 0.6 | 0.1 | 122.2 ± 1.4 | 0.161 ± 0.009 | -54.1 ± 0.3 |
| LD16M | 2.0 | 0.1 | 154.1 ± 0.6 | 0.147 ± 0.003 | -53.2 ± 2.7 |
|  | 4.0 | 0.1 | 225.3 ± 3.7 | 0.341 ± 0.012 | -42.8 ± 0.9 |
|  | 2.0 | 0.25 | 147.7 ± 0.9 | 0.149 ± 0.007 | -59.0 ±0.8 |
|  | 0.15 | 0 | 204.8 ± 3.3 | 0.314 ± 0.023 | -52.1 ± 1.9 |
|  | 0.6 | 0.1 | 126.9 ± 0.7 | 0.123 ± 0.010 | -54.2 ± 0.7 |
| LD16B0.2M | 2.0 | 0.1 | 167.5 ± 0.8 | 0.167 ± 0.018 | -54.4 ± 1.5 |
|  | 4.0 | 0.1 | 182.5 ± 5.6 | 0.299 ± 0.053 | -41.0 ± 1.4 |
|  | 2.0 | 0.25 | 159.5 ± 1.0 | 0.175 ± 0.006 | -58.4 ± 0.9 |

**Figure S1:** SEM images of PLA-based PUD particles obtained from different emulsion preparation conditions: (a)-(b) PUD-0.5(2%)-SDS(0.1%) and (c)-(d) PUD-0.5(2%)-SDS(0.1%)-NaOH(0.001M).
